# Bibliometric Properties of Placebo Literature From the JIPS Database: A Descriptive Study

**DOI:** 10.3389/fpsyt.2022.853953

**Published:** 2022-03-25

**Authors:** Katja Weimer, Cliff Buschhart, Ellen K. Broelz, Paul Enck, Björn Horing

**Affiliations:** ^1^Department of Psychosomatic Medicine and Psychotherapy, Ulm University Medical Center, Ulm, Germany; ^2^University Library, Brandenburg University of Applied Sciences, Brandenburg an der Havel, Germany; ^3^Department of Psychosomatic Medicine and Psychotherapy, Tübingen University Hospital, Tübingen, Germany; ^4^Department of Systems Neuroscience, University Medical Center Hamburg-Eppendorf, Hamburg, Germany

**Keywords:** placebo effect, bibliometrics, journal impact factor, authorship, publications

## Abstract

**Objectives:**

First dedicated articles about placebo effects have been published in the 1940s, and more than 5,000 articles have been published in scientific organs since. However, the evolution of this research field has rarely been examined. By means of bibliometric analyses we aim to generate research metrics such as the number and types of publications as well as topics, authorship networks, impacts, and future directions.

**Methods:**

Bibliometric methods were applied to the Journal of Interdisciplinary Placebo Studies (JIPS) database. It comprises around 5,000 scientific articles dedicated to researching placebo effects and mechanisms and is expanded continually through individual curation, making it a prime candidate for investigation. Web scraping was used to obtain complete article information from PubMed and Web of Science. The same information was obtained for addiction research as reference field. Analyses include a general characterization of the database as well as focus points concerning publication types (data vs. non-data articles), high-impact publications and more.

**Results:**

Analyses show that the JIPS database is a comprehensive collection of placebo publications. The development of the field is comparable to that of the comparator field and scientific publication in general. The most frequently used keywords describe populations or study design topics; the most frequent symptoms were pain, depression and anxiety. Data and non-data (e.g., review) papers are related in proportion of about 6:4 in recent decades, indicating a stable degree of productivity. A network of 26 interconnected researchers was identified who published 25 or more articles. Placebo research contributes comparable numbers of publications to high-impact journals as the comparator field. Several additional analyses are performed, with a focus on visualization of various database parameters.

**Conclusions:**

Bibliometric analyses of the JIPS database can be used to answer questions to the field, for example, to get an impression of blind spots and future directions. However, keywords used in indexing and publications themselves are often general and suggest that placebo research may still be considered a subspecialty of superordinate fields, particularly since there are no journals dedicated to placebo research itself. We invite interested colleagues to use this database for further analyses.

## Introduction

The development of a new subspecialty in most if not all areas of science and research is usually not well documented but may occur in many incremental steps in diverse scientific areas over a prolonged period of time. Usually, it can only be evaluated retrospectively after its members have established some formal and informal rules of communication. As an example, it may be quite difficult to identify and describe exactly when placebo research—that is, research dedicated to mechanisms of placebo effects, their occurrence, and related aspects—became a subspecialty of medicine, psychology and related fields. After establishing a scientific society (Society of Interdisciplinary Placebo Studies; SIPS) (2014), exchange about novel findings (e.g., the Journal of Interdisciplinary Placebo Studies—JIPS—newsletter, 2016), communication formats (e.g., the SIPS conferences starting in 2017), and consensus proposals concerning terminology and implications [2018, (1)], the current status of placebo research and researchers is, without doubt, that of a unique scientific community. While it is possible to identify when the term “placebo” entered the scientific terminology, its use and acceptance within the established communities remains largely in the dark. However, the definition of the term “placebo effect” was recently described by an expert consensus as “the changes specifically attributable to placebo and nocebo mechanisms, including the neurobiological and psychological mechanisms of expectancies” (1, p. 206). Bibliometric approaches may help to uncover this history, and structure the past and present state of this “new kid in town”.

Bibliometrics itself has—as a novel scientific discipline—a similarly “dark” beginning. It has its origins in the library and information sciences, where it first applied mathematical and statistical methods to books and other science communication media (2). Its development toward an own research area began in the 1920–1930s, when important bibliometric laws were postulated. For example, Lotka's law (3) postulates a systematic relationship of the number of few prolific vs. many onetime authors; Zipf's law (4) similarly addresses the probability of word occurrences in a given text [also see (5, 6)]; and Bradford's law (7, 8) postulates the centrality of a small number of journals in any given field, and an increasingly wide periphery.

Another key moment in the history of bibliometrics was the introduction of the Science Citation Index (SCI) in 1955 (9). A byproduct with high relevance was the Journal Citation Report (JSR) serving to identify authors, their publications, and their citation frequency (9). The first international scientific journal with specialization for bibliometrics and quantitative analysis of science products was Scientometrics, published in 1978 (10). Bibliometrics is defined as the study of quantitative structures in science, science communication, and science politics (10), with numerous related (sub)disciplines such as scientometrics or webometrics (11). Of specific importance for the discipline are publications of research results. In empirical research, this mostly includes printed journal publications—as compared to the eighteenth and nineteenth century dominance of science books –, and more recently online publications (12, 13). Bibliometrics mostly uses scientific articles published in specialized journals as its dominant research subject. In this paper, we will apply bibliometric approaches to study placebo research.

The term “placebo” was coined in the eighteenth century (14), and seminal, dedicated, mechanistic placebo research papers have been published as early as 1946 (15). A first bibliometric analysis (16) of placebo papers was limited to 301 published papers, while the number of genuine papers in the JIPS database had already increased more than ten-fold by 2015 (17). As of 2021 it comprises nearly 5,000 papers containing data-based publications, reviews, and meta-analyses.

This study aims to cover a broad range of analyses and visualizations to characterize the JIPS database; in particular, it aims to:

i) ascertain quality and basic content of the JIPS database and its comparator.
ii) elaborate on the content through keyword frequencies and author networks.
iii) quantify the importance and productivity of placebo research compared to all publications in specific fields such as pain, depression, or anxiety.
iv) describe performance aspects of placebo literature (e.g., impact, receptivity) compared to publications in a comparator field (addiction research).

Bibliometric methods are myriad and differ in their degree of sophistication, and most parameters (e.g., frequency, impact) carry substantial caveats (18, 19). As this is a fledgling and ongoing project, it will limit itself to some aspects that might prove helpful for the placebo research community at this stage.

## Materials and Methods

### Origins of the Database

The creation of the Journal of Interdisciplinary Placebo Studies (JIPS) database has been described elsewhere [e.g., (17, 20–22)]. Briefly, in 2004, PE started to collect all research articles dealing with the placebo effect by searching the PubMed database retrospectively and prospectively using the simple descriptor “placebo” (All Fields). Since then, new entries in the PubMed database are being curated on a weekly basis for placebo research articles by a team of researchers (PE joined by KW and EKB). Articles are added to the JIPS database (administrated by BH). It has been made available for the interested public in 2016 (https://jips.online).

### Preprocessing

The JIPS database is administrated in the citation manager software EndNote (23). To obtain a dataset suitable for analysis, the database underwent several steps of preprocessing. First, the database obtained from EndNote (status as per 2021-10-11) was imported into MATLAB (version 9.8.0.1417392 (2020a); The MathWorks Inc, Natick, Massachusetts, USA). All following steps were performed in MATLAB.

Articles without PubMed ID (PMID) were identified and the PMID manually researched and entered if available. Forty-six of 128 articles without PMID could be completed, the remaining 82 articles include periodicals or monographs not listed in PubMed. No content classification was performed for articles without PMID; likewise, content classification was used as-is (without further validation or self-classification where no MeSH terms were provided). After completion, the database was double-checked for duplicates, yielding *N* = 4,895 articles in total, of which 4,732 articles have a valid PMID.

In a next step, to ensure up-to-date information, all PMIDs were extracted and used to re-query all available information from two sources: PubMed (https://pubmed.ncbi.nlm.nih.gov, United States National Library of Medicine, National Institutes of Health) and Web of Science (WoS; https://www.webofscience.com, Clarivate Analytics). This information was appended to the database. While mostly redundant, it includes data not commonly available in EndNote, such as full author names, Medical Subject Headings (MeSHs), or citations counts.

Next, all authors were compiled and uniquely identified by using full names. Authors whose full names were not provided, and where abbreviated names were ambiguous, were manually identified if possible.

### Derived Data Sets

For each article in the database (subsequently called parent articles), information about all citations (i.e., articles citing the parent) were downloaded from PubMed using web scraping functions provided by MATLAB. Specifically, the Cited By-field in PubMed's result pages were looped and parsed to identify citations information. Analyses involving citations are therefore further restricted to PubMed-provided data only.

For further analyses concerning data and evidence types, the PubMed field Publication Type was used. Articles were either defined as “data” or “non-data” articles depending on their publication type (Supplementary Figure 1). Publication types tagged as “uninformative” were not considered in the respective comparative analyses.

For the existing datasets, the units of analysis employed were:

- articles (commonly by Pubmed ID, PMID)
- authors
- references (i.e., articles cited in an item of the database)
- citations (i.e., articles citing an item from the database).

### Comparator Database

After initial analyses, it became apparent that not all were informative in isolation but required a comparison with similar data sets outside the placebo field. For example, to know whether the ratio of first authors to all authors in the placebo field was somehow remarkable it has to be compared to a similar field. For the current analysis, we decided on addiction research as a comparator. Importantly, the field includes highly interdisciplinary approaches, the clinical entity is also signified by a large psychological component, and the number of publications is roughly comparable to that in placebo research (or at least not different by orders of magnitude). Web scraping was used to search PubMed for the MeSH Major Topic “behavior, addictive” (alias “addiction”; MeSH Unique ID D016739). All steps described above were also taken for this single-descriptor database.

### Medical Subject Headings and Other Indexed Parameters

Several of the following analyses rely on MeSH descriptors provided by PubMed. MeSHs are a controlled vocabulary thesaurus administrated by the National Library of Medicine (https://www.nlm.nih.gov/bsd/indexfaq.html). The thesaurus includes over 27,000 entries which can be qualified with over 80 subheadings. While it is occasionally expanded by new terms, these are not exhaustively applied retroactively to already indexed articles. Similar to MeSH indexing, PubMed provides fields concerning, for example, the publication type of an article. All indexing is performed by trained indexers. MeSH terms are routinely subject of bibliometric investigations (24, 25), although possible misclassification is a concern to be addressed.

### Analysis Strategy and Caveats

The focus of this manuscript is on description and visualization of database contents, with only few analyses deemed to profit from inference statistical approaches. For these analyses, the significance level was set to *p* = 0.05.

Wordclouds were generated using the MATLAB function wordcloud and including the 100 most frequent terms in the list of all MeSH terms used in the classification of the JIPS database. The words “placebo” and “placebo effect” were excluded because they constitute the selection criterion for inclusion in the JIPS database to begin with. Author networks were created using MATLAB's graph object.

Journal impact factors were downloaded from the Scopus Database (Elsevier, Amsterdam, Netherlands). Journal aliases were downloaded from WoS.

We have opted to include all data including from the (then) ongoing year 2021 regardless of possible lags in classification, to convey as complete a picture as possible, and to preserve the same time frame between analyses. Where this would impact interpretability of recent results, a cautionary note has been added. However, where parameters cannot be computed otherwise (e.g., *n*-year impact factors), data has been truncated accordingly.

Furthermore, some analyses that rely on MeSH classification come with the caveat that the terms for “placebo effect” (MeSH ID D015990) and “behavior, addictive” (MeSH ID D016739) were introduced in 1990 and 1992, respectively. While some retrospective classification has been performed, this is largely deficient, therefore, the introduction date of 1990 has been added as a visual marker/cautionary note where appropriate.

## Results

### Database Volume and Integrity

Our first intention was to ascertain quality and integrity of the JIPS and comparator databases, and provide a general overview.

At the reference date (2021-10-11), the JIPS database included 4895 unique entries, 4110 of which include attached documents. Of these, 4723 are PubMed-listed entries, meaning 172 articles (3.5%) are not listed; note that these articles are not considered in most analyses. The number of detected citations was 36,631.

At the reference date, the addiction database included 8,986 unique PubMed-obtained entries. The number of detected citations was 52,782. The overlap between the JIPS and comparator database was negligible (1 article only, PMID 15361811).

**Figure 1:**
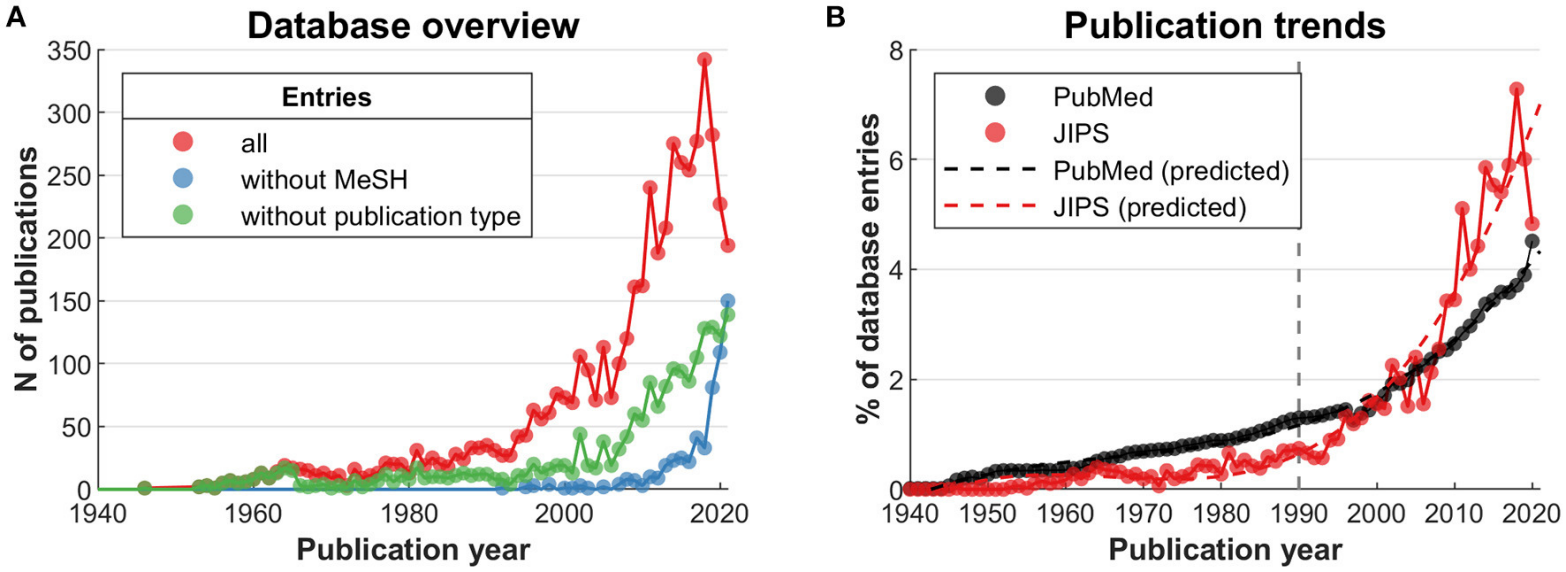
JIPS database overview compared to all PubMed publications. The gray vertical line marks the introduction of the “placebo effect” MeSH term (1990). **(A)** Number of all publications in the JIPS database (red line). Of these, a number of publications have not been MeSH-indexed at the time of data compilation (blue line), with a higher number in the most recent years. Likewise, a number of publications do not have an informative Publication Type (green line). See text for details. **(B)** Publications in the entire PubMed database compared to the JIPS database, normalized by total database volumes. The JIPS trajectory (red dashed line) first undercuts the PubMed trajectory (black dashed line), then exceeds it around year 2001. Predicted values from random intercept linear mixed effects model with quadratic term.

The red line in Figure 1A shows the number of publications by publication year starting at 1940, with the first JIPS entry occurring in 1946 (15). The increase is roughly proportional to the one seen for the entire PubMed database in this time frame (Figure 1B). More specifically however, while placebo research was relatively less productive prior to the mid 1990s, recent years have seen an increase in publications exceeding that of the general scientific output, as indicated by the dashed lines (year × year^2^ × database interaction, *t* (154) = 5.222, *p* = 6 × 10^−07^; random intercept model including linear and quadratic terms).

The MeSH- and publication type-based analyses in this work rely on the correctness and completeness of indexing. To investigate these aspects, we first determined the number of non-indexed publications in the JIPS database. For MeSH terms (Figure 1A, blue line), indexing shows a conspicuous lag. Presumably, this is because of the backlog involved in consecutive indexing given an ever-increasing number of publications (Figure 1B). The number of articles without an informative publication type (Figure 1A, green line) shows a lag as well, but an even higher number of affected articles. For a determination of informative vs. noninformative publication types, see section “Productivity in the context of parent fields and data generation” below.

As for the question of whether MeSHs accurately describe an article's content if indexing was performed, cursory analysis indicates that they are not applied with full consistency. For example, 857 articles use “pain” or “analgesi^*^” in the abstract, 915 articles use “pain” or “analgesi^*^” in the MeSHs. However, of the 857 abstract hits, 183 (21%) do not have the corresponding MeSH entry, whereas of the 915 MeSH hits, 241 (26%) do not use the corresponding term in the abstract. For “depression” or “depressive”, a similar ratio arises.

As a sensitivity analysis, we obtained a Major MeSH-derived dataset using the term “placebo effect” through PubMed web scraping, in analogy to the procedure used to obtain the addiction comparator database. This dataset contained 2,174 entries. We determined the intersection to JIPS using PMIDs—with 1,471 articles in both datasets, 703 articles were identified by the MeSH but not by the JIPS curation. These 703 were processed in their entirety and judged by abstract inspection whether or not they should be included in the JIPS. Two hundred and fifty articles were considered to be pertinent, indicating “misses” in the range of 250/(4,895 + 250)≈5%. While these articles were added to the JIPS going forward, we decided to proceed with the status quo in this paper to preserve continuity to the previous JIPS-based publications (17, 20–22). Note that conversely, the PubMed classification only identified about a third of articles contained in the JIPS database (1,471+250)/(4,895+250)≈33%. This issue also has implications for the comparison between JIPS and (Major MeSH-derived) addiction databases that are discussed under “Performative aspects and comparative database analysis”.

### Content Characterization and Authorship Networks

**Figure 2:**
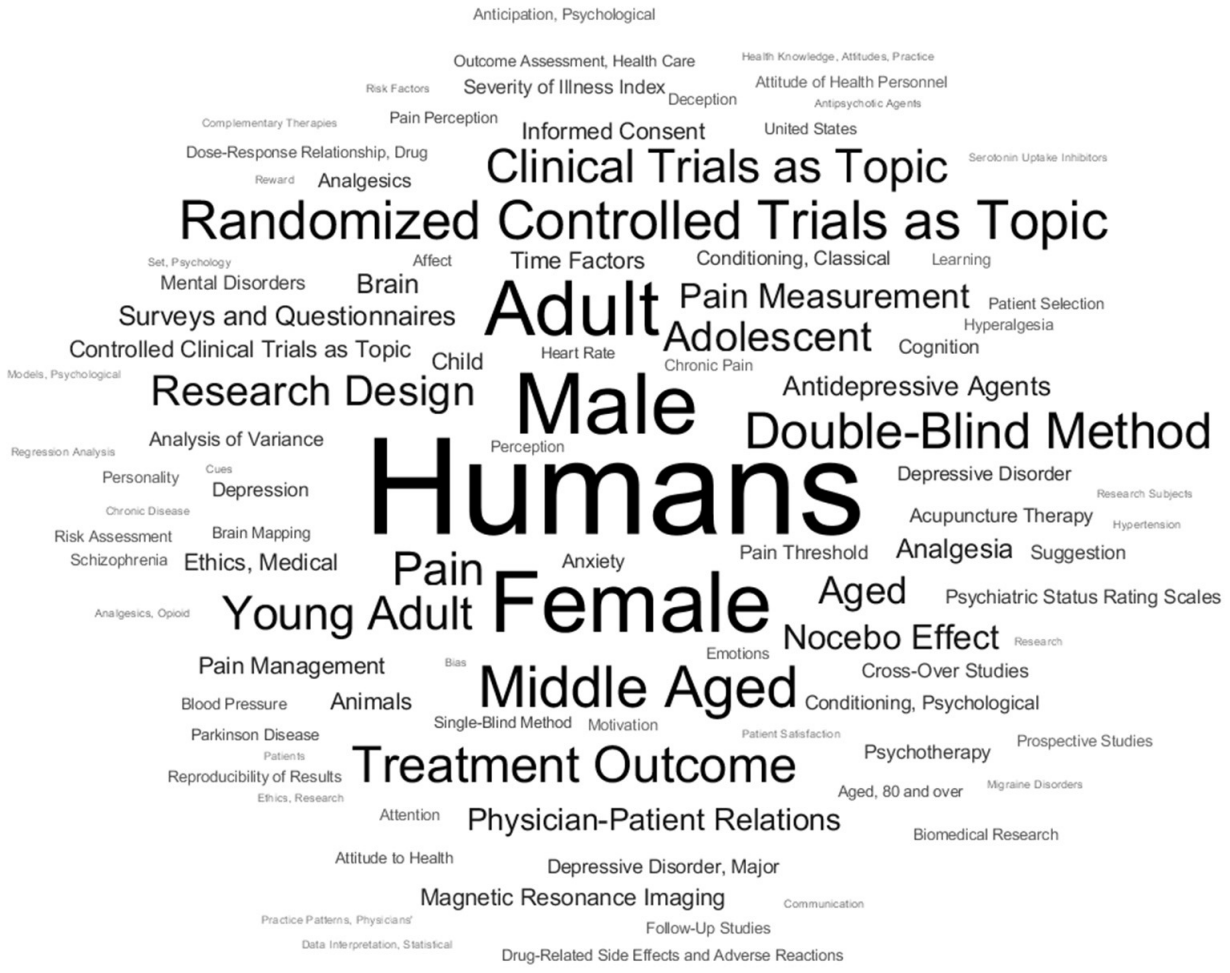
Word cloud of (major) Medical Subject Headings (MeSH) used in the JIPS database. Larger words indicate more frequent occurrence.

**Figure 3:**
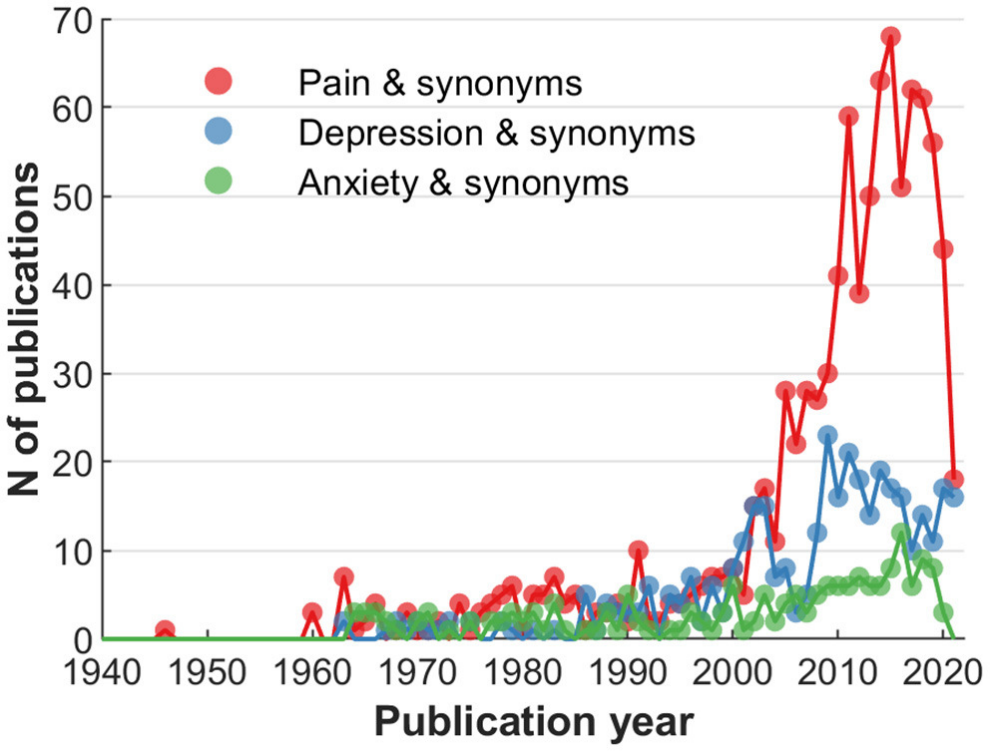
Comparative frequency of articles by MeSH terms: Related to the investigated entity (e.g., clinical symptom).

The first exploration of the actual JIPS database content included the MeSH terms employed, and collaborative networks of its contributing researchers. To better convey the contents of available MeSHs, a compilation of the most frequent occurrences is shown in Figure 2. Broadly, the most frequent terms can be categorized into generic (e.g., placebo effect, treatment outcome), population-related (e.g., humans, adult, female, age), design-related (e.g., randomized controlled trials as topic, double-blind method), entity-related (e.g., pain, depressive disorder) or measurement-related (e.g., pain measurement, brain). As an example for entity-related terms, Figure 3 shows the frequencies of the top three symptoms investigated (and indexed) in the JIPS database, namely pain, depression and anxiety including synonyms. Decreases are likely due to similar reasons as the general drop in publication numbers discussed concerning (Figure 1).

**Figure 4:**
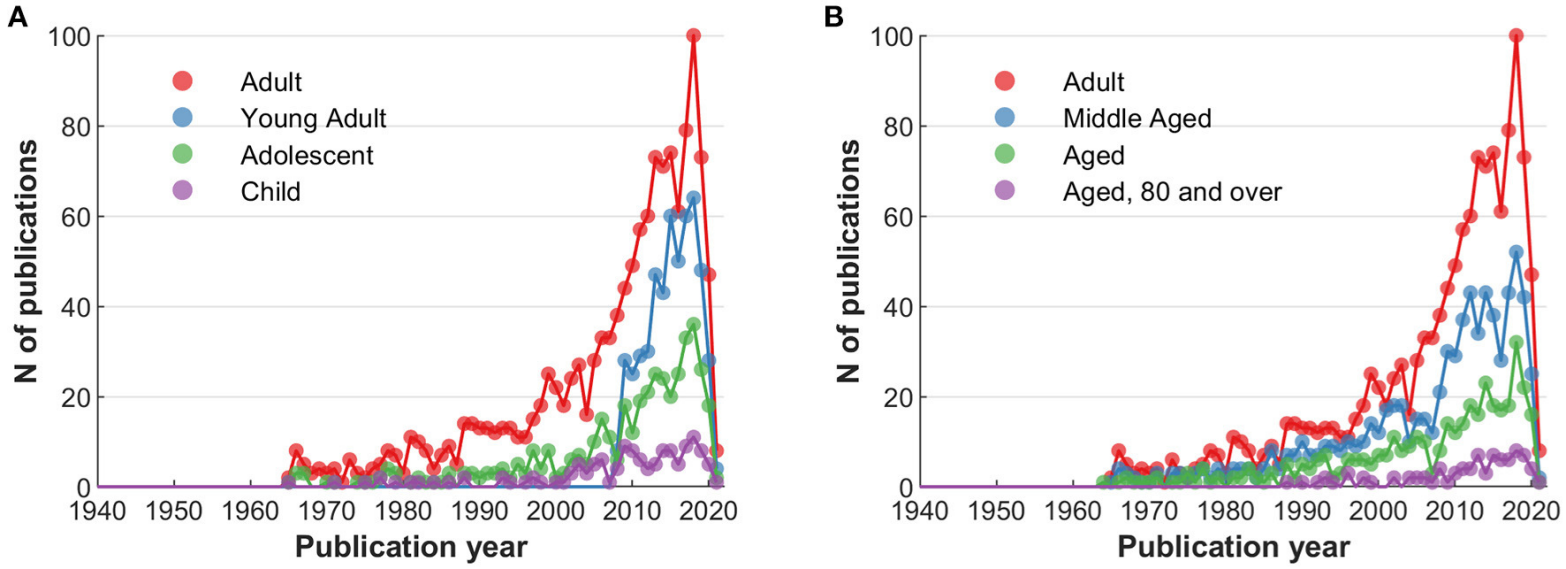
Comparative frequency of articles by MeSH terms: Related to the investigated population. **(A)** Younger ages, with “adult” MeSH as reference. **(B)** Older ages, with “adult” MeSH as reference.

Since population-related terms were among the most frequent, it is worthwhile to assess MeSHs relating to age distributions to consider possible regularities or even shortcomings (26). Figure 4A shows the frequency of articles investigating younger populations (child, adolescent, and young adult), Figure 4B that of older populations (middle aged, aged, over 80 years of age); the “adult” MeSH is provided as reference in both.

**Figure 5:**
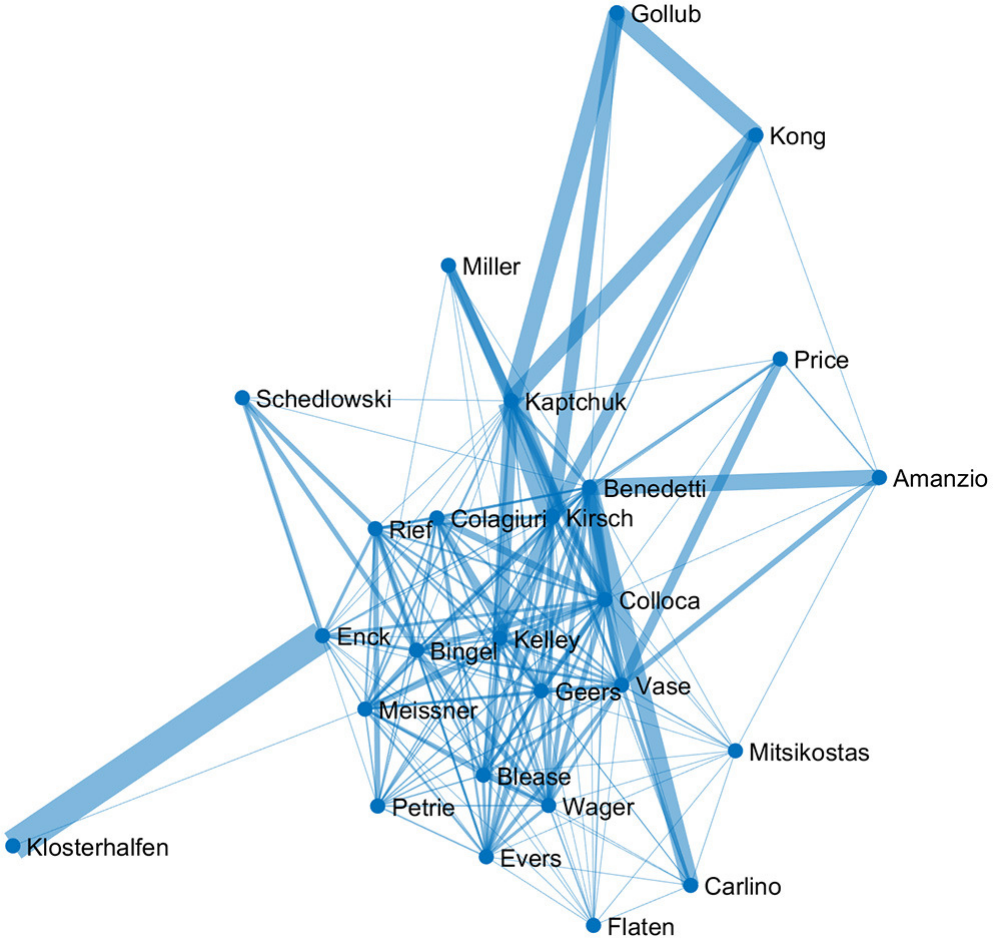
Network of the most prolific authors in the JIPS database. Cutoff criterion for inclusion were ≥25 publications included in the database. Line width indicates the weight of the connection, corresponding to the number of shared publications. Centrality indicates the number of interconnected researchers.

As a final illustration of basic information contained in the database, co-authorship data can be processed to show collaborative networks between individual authors (Figure 5). This not only allows for an assessment of collaboration strength (for example, particularly strong collaborative relationships exist between Enck and Klosterhalfen, or Kaptchuk and Kirsch), but also the interconnectedness of the respective authors (for example, Gollub and Klosterhalfen are located on the periphery; conversely, Bingel and Geers have ties to a larger number of collaborators).

### Productivity in the Context of Parent Fields and Data Generation

**Figure 6:**
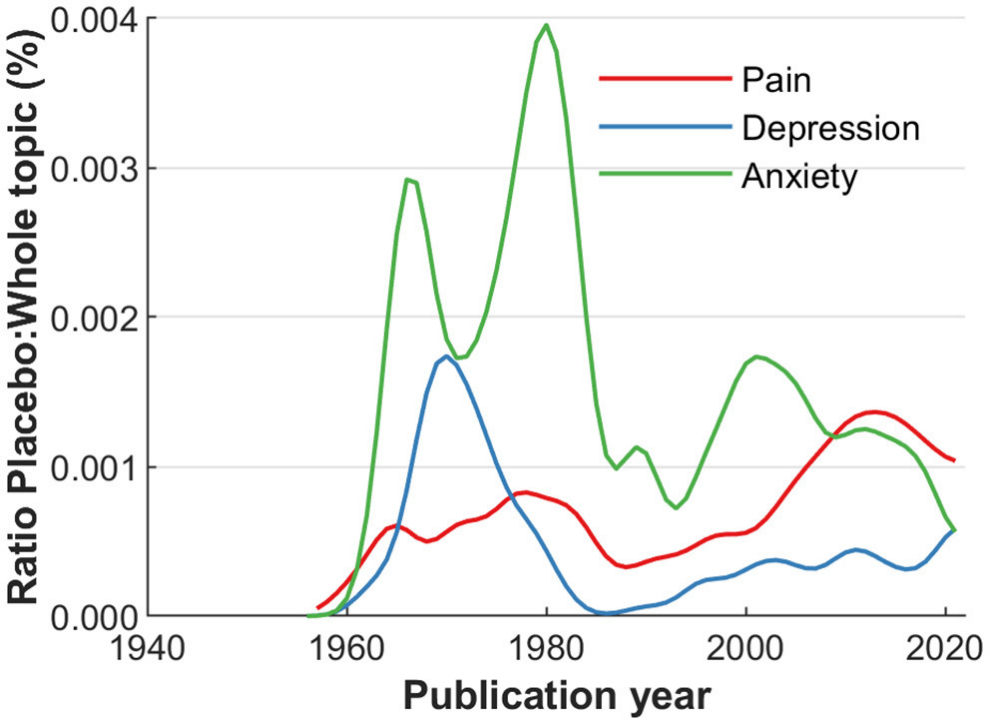
Major MeSH term occurrence in the database, compared to the number of citations in the entire respective PubMed field (pain, depression, anxiety) over time. Absolute percentages are low, but allow to identify peaks of interest and trends in the respective fields. Data is smoothed over 5 years.

Additional information about the placebo field can be garnered using more sophisticated analyses on the JIPS database by relating placebo-related information with those available for broader fields in which placebo-related research takes place. For example, Figure 6 plots the ratios of major entity-related entries in the JIPS database (pain, depression, anxiety) in relation to the entire number of publications in the respective field. This analysis reveals two types of information. Firstly, that the proportion of placebo-related information in any given field is diminutive, as indicated by the low percentages shown by the graphs (around 0.001%, i.e., one in hundred thousand articles being dedicated placebo research). Secondly, the trajectories can be used to identify trends in the involvement of placebo research in any given field, keeping in mind that small base rates of placebo publications lead to a higher volatility in the respective curves (e.g., anxiety). For example, relative to the entire number of publications in depression research, placebo-related studies peaked around 1,970 and recently started increasing again.

As another example, the PubMed-provided field Publication Type (including one or more entries per article) can serve as a diagnostic tool to assess the generative power of the placebo field, e.g., by illustrating the ratio and composition of articles containing original (experimental or clinical, people-derived) data vs. derivative articles such as reviews or meta-analyses. For this purpose, the publication types from the JIPS database were compiled and divided into either category (“data” or “non-data”, see above). A full list of categorized publication types is found in Supplementary Figure 1. For example, “data” publication types include those tagged “Classical article”, “Clinical study” or “Observational study”; “non-data” publication types include those tagged “Editorial”, “Review”, “Meta-analysis”. A third category was established as “uninformative”—these publication types were not included for analysis as they do not discriminate between data or non-data articles (e.g., “Journal article”, “Research support, non-US gov't”, “English abstract”). Articles were categorized as data, non-data or uninformative in a hierarchical fashion, i.e., where multiple tags were present, data tags had precedence over non-data tags; uninformative tags were removed altogether.

**Figure 7:**
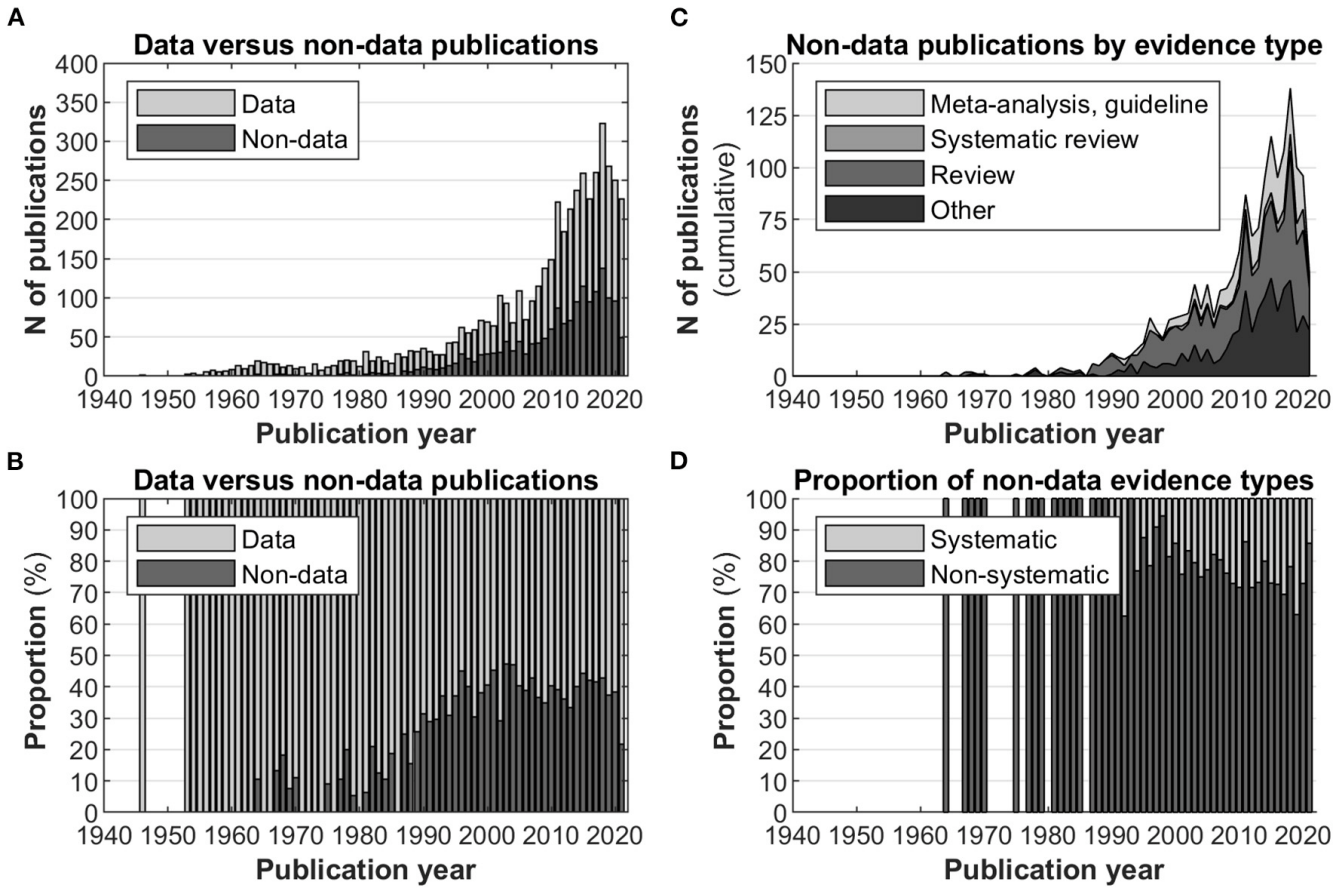
Data vs. non-data publications, excluding uninformative publication types. **(A)** Histograms of data articles vs. non-data articles in the database. **(B)** Proportion (in percent) of data vs. non-data publications. In recent years, this ratio is relatively stable, with the majority of the articles considered data publications. **(C)** Breakdown of the evidence types of non-data publications. **(D)** Proportion of non-data evidence types categorized in non-systematic or systematic. Systematic evidence types are increasingly utilized.

Figure 7A displays the number of non-data publications in the database in relation to the total number of publications (also see Figure 1A); Figure 7B displays the ratio between the two categories, indicating a relatively stable proportion of ca. 40% non-data papers in the past 25 years.

Relatedly, the quality of evidence provided by non-data articles can vary [e.g., (27)], with studies including aggregate statistics such as meta-analyses providing the highest level. The exact criteria for this subdivision are provided in Supplementary Table 1. Subdividing the non-data papers into categories of evidence quality, Figure 7C displays the relative frequencies, with narrative/non-systematic reviews constituting the bulk of non-data publication types. Further aggregating evidence types, Figure 7D demonstrates that the level of systematic evidence types remained relatively stable in the past 25 years, albeit a slightly increasing trend is discernible.

### Performative Aspects and Comparative Database Analysis

**Figure 8:**
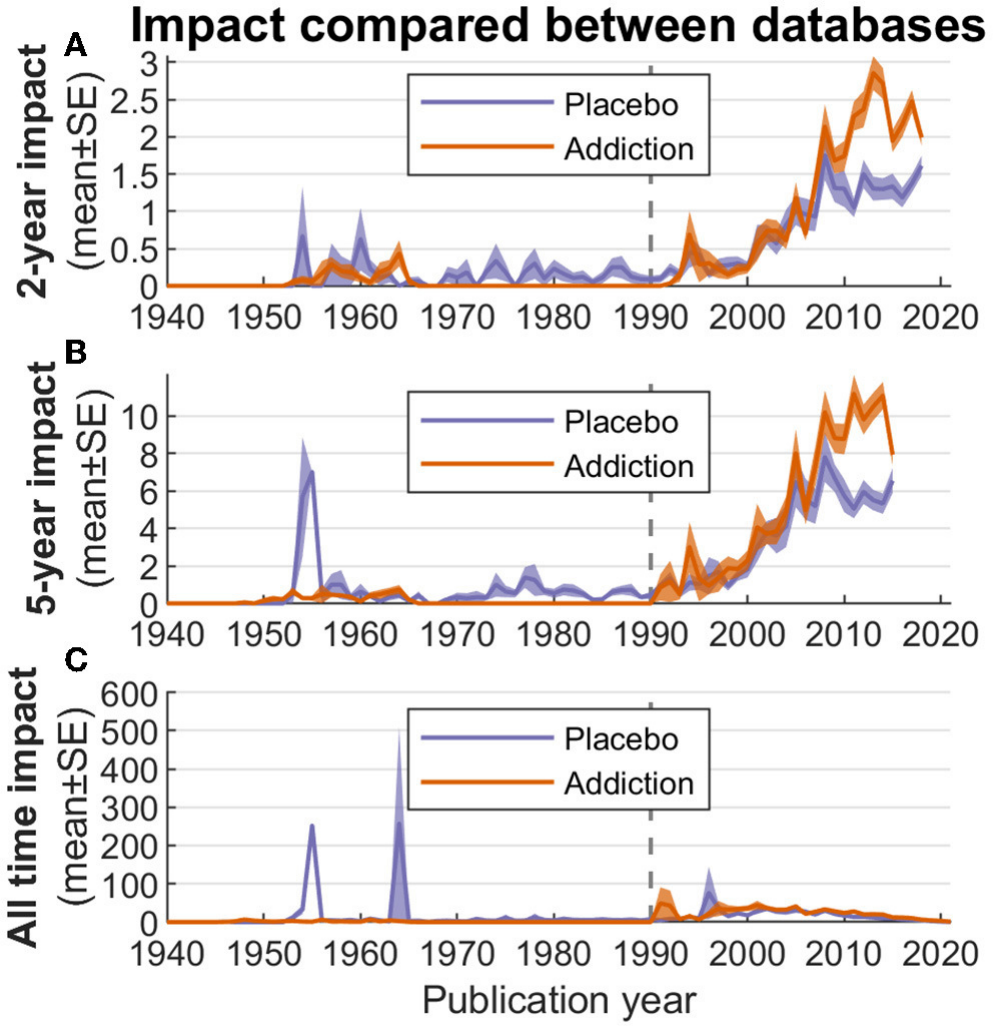
Average impact of all articles per year in the JIPS database and comparator database. **(A)** 2-year impact. **(B)** 5-year impact. **(C)** All-time impact. The gray vertical line marks the introduction of the “placebo effect” MeSH term (1990), coinciding closely with introduction of the “behavior, addictive” MeSH term (1992).

To further characterize the field, we considered performance aspects such as impact, reception parameters, and qualitative aspects of individual high-performing publications. These analyses were contextualized with data from our addiction comparator database. For example, Figure 8 shows the average impact of all articles of placebo vs. addiction research. For the 2- and 5-year impact factors, the comparator database outperforms placebo research in the past 10 years. Comparing the all-time impact (Figure 8C) with the more constrained alternatives, it is also possible to detect seminal papers by identifying “spikes” in the impact parameter (see Table 1 below).

**Table 1:** Articles with highest age/citation ratio.

**PMID**	**First author**	**Title**	**Year**	**Journal**	**Total ***N*** of citations**	**Mean ***N*** of citations per year**
13271123	Beecher	The powerful placebo	1955	J Am Med Assoc	252	3.8
24141714	World Medical Association	World Medical Association Declaration of Helsinki: ethical principles for medical research involving human subjects	1964	JAMA	5,071	87.4
80579	Levine	The mechanism of placebo analgesia	1978	Lancet	194	4.4
8721797	Jadad	Assessing the quality of reports of randomized clinical trials: is blinding necessary?	1996	Control Clin Trials	4,332	166.6
9250266	Bucher	The results of direct and indirect treatment comparisons in meta-analysis of randomized controlled trials	1997	J Clin Epidemiol	553	22.1
9252330	Rainville	Pain affect encoded in human anterior cingulate but not somatosensory cortex	1997	Science	524	21
12649484	Fiorillo	Discrete coding of reward probability and uncertainty by dopamine neurons	2003	Science	647	34.1
14976306	Wager	Placebo-induced changes in FMRI in the anticipation and experience of pain	2004	Science	511	28.4
15995724	Vogt	Pain and emotion interactions in subregions of the cingulate gyrus	2005	Nat Rev Neurosci	635	37.4
16100511	Harris	A role for lateral hypothalamic orexin neurons in reward seeking	2005	Nature	481	28.3

*PMID, Pubmed ID*.

**Figure 9:**
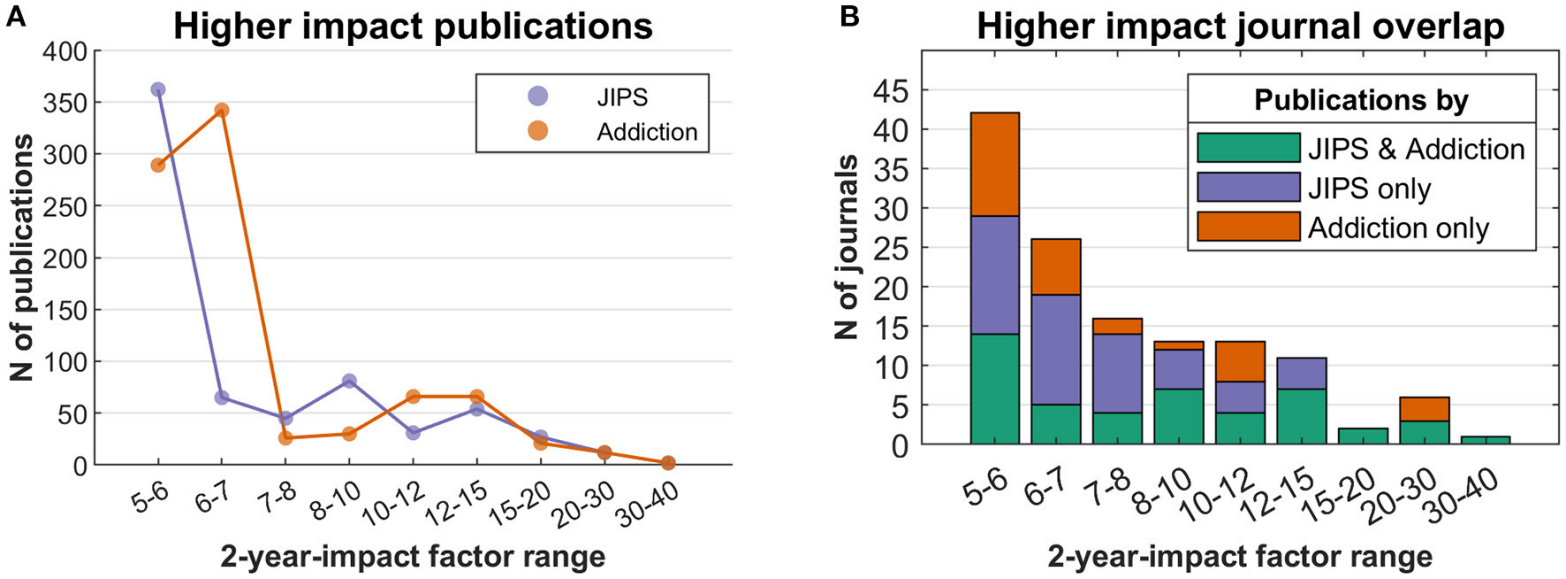
Publications in high-impact journals in JIPS database and comparator database. **(A)** Number of publications in journals binned by 2-year impact factor. **(B)** Overlap of journals in which either database publishes its results. Stacked bars indicate shared journals (bottom), JIPS-only journals (middle) and comparator-only journals (top).

Figure 8 also indicates that articles in the JIPS are cited less frequently than the comparator database in (roughly) the past 10 years. In an analysis focusing on journals in upper impact segments (Figure 9A), we have determined that no clear distinction emerges between JIPS and the comparator database, i.e., both publish in comparable quantities in higher-impact journals; note that this is despite the comparator database containing roughly twice the number of publications. However, the lower two (~medium) impact factor bins (factors 5–6 and 6–7) hint at an overall advantage of the comparator database in these journals, which is driven by a journal dedicated to that field (312 publications in the Journal of Behavioral Addictions, impact factor 6.21; see Supplementary Table 2). In an adjunct analysis, we can demonstrate the overlap between the two fields in terms of journals they publish in (Figure 9B)—of 130 journals used by either, 47 are used by both (36%) and 83 separately.

**Figure 10:**
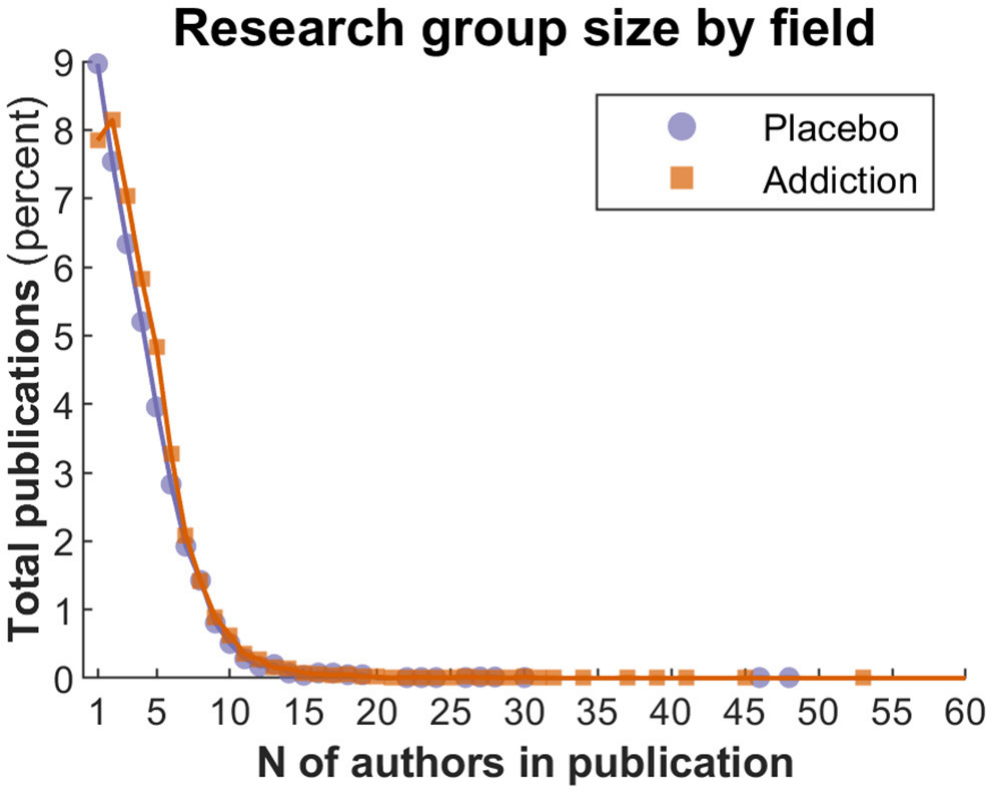
Research group size by field.

Figure 10 shows the average number of authors in a publication, by field (placebo vs. addiction). The percentages are broadly comparable, with an initial difference between the two fields, such that in placebo research (as per JIPS database), more single-author publications are registered.

**Figure 11:**
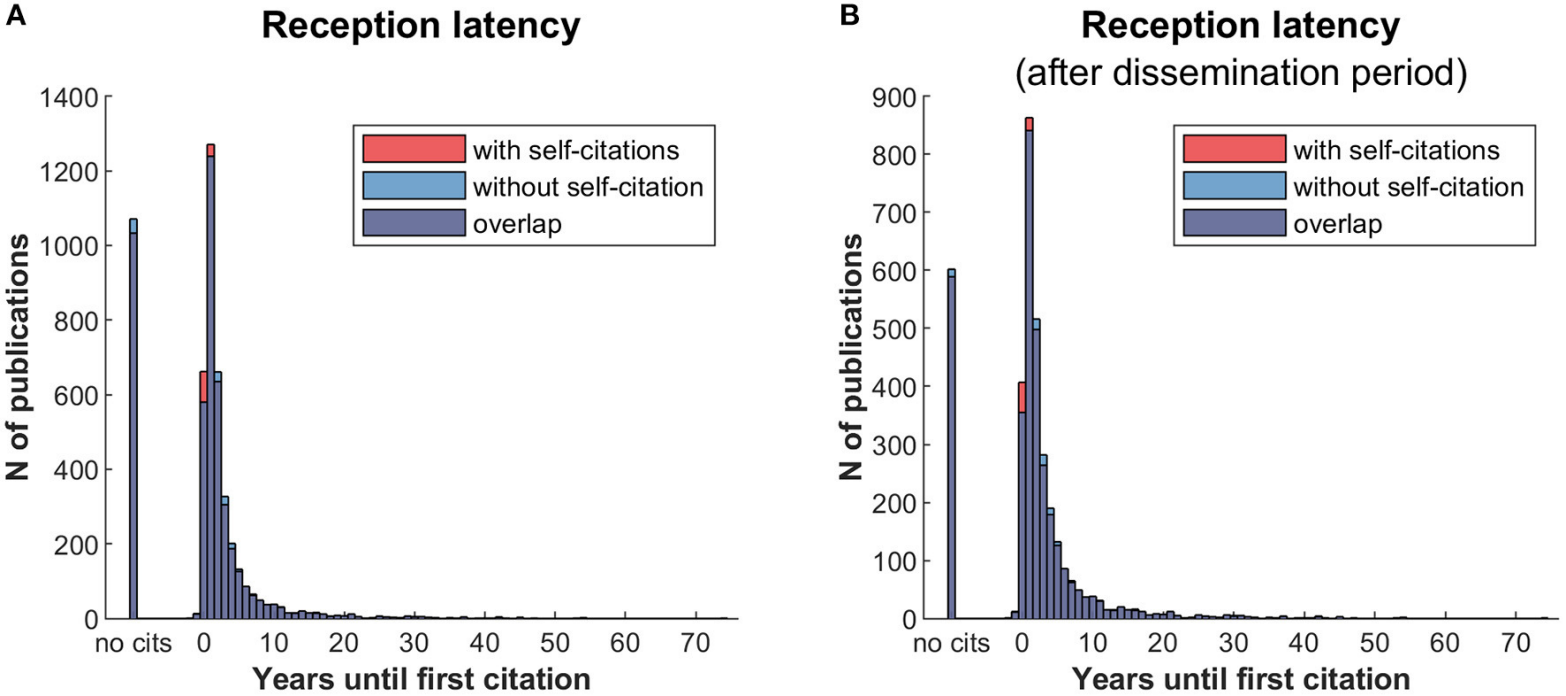
Reception latency. **(A)** Years until first citation for all JIPS articles. **(B)** Years until first citation including dissemination latency of 5 years, i.e., including only articles aged 5 years and older. Results resemble those from A, but with (expectedly) fewer non-cited articles.

Figure 11A shows the latency with which new publications are cited. Peak latency occurs after one year, at which time around a quarter of articles were cited (~1,300).

**Figure 12:**
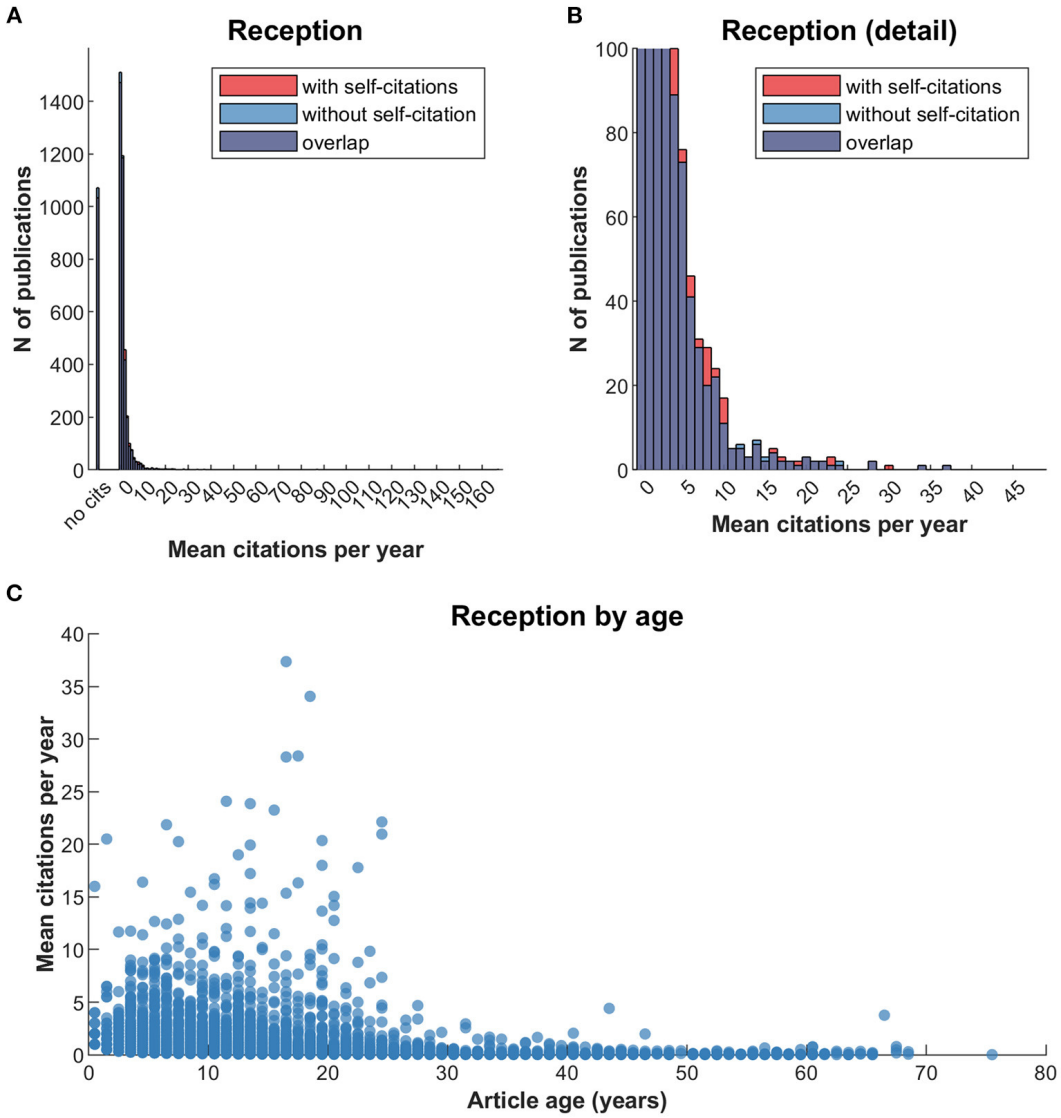
Reception of JIPS articles over time. Note that this figure is limited in that article age and its dissemination are not unrelated, because very young articles may have had insufficient time for reception. **(A)** The number of publications binned by mean citations per year. **(B)** Same data as **(A)**, but with adjusted y axis to see details of the distribution more clearly. **(C)** Mean citations per year, by article age. For illustration purposes, axes were set to exclude two very high-performing articles (PMID 24141714 with age 57 and 87.4 mean citations; PMID 8721797 with age 24 and 173.3 mean citations).

Figure 12 illustrates the average success of the JIPS articles over time. Like Figure 11A, Figure 12A shows that roughly a quarter of articles in the database (~1,100) are not cited. However, this includes the fact that the database used for these analyses include a high number of very recent articles (e.g., 282 from 2019, 227 from 2020, 194 from 2021) which may not have been sufficiently disseminated, or whose citations have not yet been published. Most articles included in the database are only cited once per year after publication, with rapid decreases in frequency as the number of citations increase.

Figure 12C plots the age of an article against its average citations per year. This illustration is useful to detect “high performing” articles that rise above the average reception of articles of the same age. Note that the distribution is necessarily left-leaning, as the average citations per year decay at a set rate (x/year, where x crucially depends on the size of the field, which constitutes the upper bound of article reception).

Next, we compiled the 10 highest performing articles from Figure 12 in Table 1. The list not only includes seminal papers of placebo research (e.g., Beecher 1955, Levine 1978), but also general method-related (e.g., Jadad 1996) or entity-related articles (e.g., Rainville 1997 for pain).

**Figure 13:**
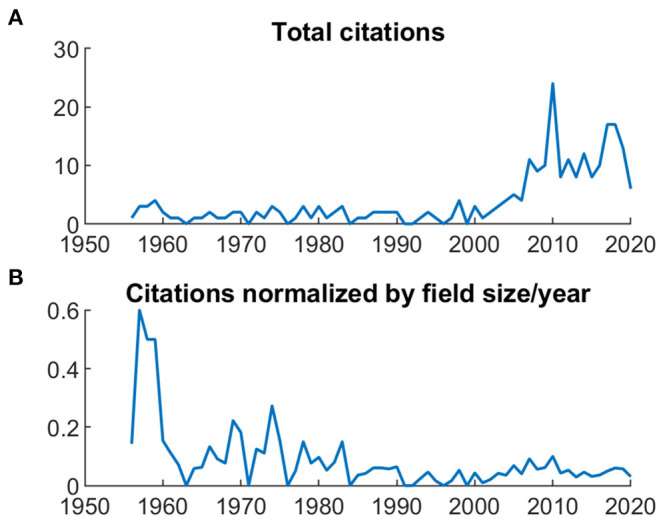
Citation trajectories for an exemplary high-performing article (Beecher 1955). **(A)** Total citations. **(B)** Citations normalized by field size (i.e., number of articles published in the respective year).

In Figure 13, the trajectory of a seminal paper (Beecher 1955) is traced since publication. While panel A shows an increase in citations since around 2000, normalizing the citation rates by field size (i.e., total number of entries in the JIPS database; panel B) shows that in the years following publication, the article was cited in roughly every other publication.

## Discussion

We applied bibliometric analyses to the JIPS database and its comparator (addiction) database with several related aims. These included i) ascertaining quality and basic content, ii) elaborating database content with keyword frequencies and author networks, iii) quantifying placebo research contributions and generativity, and iv) describing performance aspects of placebo literature compared to publications in the comparator field.

Concerning basic database content (volume), the decrease in recent years (2019 through 2021) could be owed to several factors, including lags in indexing (28) (possibly related to the COVID-19 pandemic) or the fact that as per cutoff date, the year 2021 was not yet over. Nevertheless, the placebo field seems to develop at an increased pace compared to the general scientific output as per PubMed. Notably, the sensitivity analyses comparing a general PubMed/MeSH-classified search points to some oversights made during curation; simultaneously however, it reinforces the approach taken for the JIPS by establishing it as a decidedly more comprehensive collection than using the classified search alone, which identifies only about one third of articles included in the JIPS.

Elaborating on the content, the three most frequent terms regarding symptoms are pain, depression and anxiety, and their respective synonyms (Figure 3). The most frequently studied populations are young and middle aged individuals, with decreasing number of articles at lower and higher age. These findings could indicate a potential (or even a responsibility) to explore placebo effects in other symptoms and age strata. Furthermore, bibliometric analyses provide the possibility to depict networks of researchers and their collaborations. Our analysis identified 26 researchers who published at least 25 or more articles. Even with this limited number, regional clusters can be fairly easily distinguished, as is expected from increased likelihood of collaborations due to funding mechanisms, conference attendance, if not simply geographical proximity (29).

One surprising finding is that the relative contributions of placebo publications in superordinate research fields such as pain, depression, and anxiety, are very low (below e.g., 0.0015%). Therefore, the large majority of research in placebo-affine fields (e.g., pain) stems from other sources, while dedicated placebo research plays an overall minor research role in the respective field. However, there seems to be a small increase in the interest of placebo research in these fields since the 1990s. Using terms from other MeSH categories, e.g., treatment-related methods, could be used to assess the extent to which a field draws on placebo-related treatment mechanisms. The placebo effect plays a role in every diagnostic study and in treatment effects independent of the methods and symptoms investigated, but is obviously not always recognized. On the other hand, many placebo publications deal with basic science investigations in healthy volunteers or pilot studies with patients, and large clinical studies about harnessing the placebo effect in clinical practice are—still—lacking (30). Although it is not a direct measure, our finding strongly indicates that there is room for a broader application of insights derived from placebo research.

Even a cursory glance reveals a quickly growing number of placebo publications, particularly after 1990. One concern here is that as the body of literature grows, derivative works also increase in number, to the point where a field does not generate original data anymore. This concern seems unfounded for placebo research, as the proportion of data papers to non-data papers has reached a steady state in recent years. The field's productivity is therefore relatively stable, which is an important indicator for researchers who are considering to engage with it.

Albeit interesting by itself, the performance of a research field (quantity, quality and “vitality”) cannot be judged fully without the comparison to a reference, i.e., a control group. Here, we chose publications about addiction as a reference since both research fields are interdisciplinary, deal with psychologically codetermined entities, and show similarities in their size and development over time. As we have shown (Figures 8, 9), placebo articles were published in a comparable fashion to addiction articles in high-impact journals. Overall however, addiction publications showed higher performance regarding impact factors. Whether this switch indicates a general loss of impact of placebo research, a more restricted loss of interest on the side of the superordinate fields, a higher inclination of high-impact journals to publish placebo research, or other factors, will have to be established by futures analyses.

The analysis of the reception latency indicates rapid dissemination of the majority of articles in placebo research, and the exclusion of self-citations has only a negligible effect. Allowing for a longer dissemination period by excluding articles younger than 5 years of age (Figure 11B), the pattern remains almost identical with a slightly lower proportion of non-cited articles. Table 1 shows that placebo research as compiled in the JIPS database is a highly interdisciplinary field whose contributors are rarely dedicated to this single topic. Instead, placebo research happens at the interface to treatment modalities, clinical entities or psychological mechanisms. Relatedly, it appears that Bradford's law (7, 8) positing a core set of journals in any given field, cannot be applied to placebo research at this stage, as there are no journals specifically dedicated to (or at least predominantly engaged with) this research topic. Nevertheless, certain journals have published a relatively large number of placebo publications (e.g., Pain, see Supplementary Table 2).

The performance of single articles can be quite informative about the progression of a field; here, we used one of the first articles of Henry Beecher in 1955 as an example (31). This example shows that the absolute number of citations can increase over time, but the relative number of citations compared to all publications in the field can decrease, i.e., the relevance of this article diminishes over time.

### Limitations

Limitations of the scope of the present study apply to both the JIPS database itself as well as to the Pubmed data available for analysis. Every inclusion in the JIPS database is explicitly curated and sometimes depends on factors (including possible selection biases) and criteria which exceed those of a strictly MeSH-guided algorithm, as demonstrated in the sensitivity analysis described above. Conversely, a MeSH-guided search strategy may partially fail when MeSH terms change over time, e.g., are added or removed, and may required both approaches, at least for the purpose of such bibliometric analyses as ours. Additionally, we may miss articles that are not listed in PubMed. We therefore ask our newsletter recipients and colleagues to send us newly published articles to include them in our database.

For this analysis, we have opted for a restriction to PubMed for the citation analyses for two reasons: 1) the JIPS database itself is mostly based on input queried from the PubMed database, and 2) PubMed uses a simple URL interface and provides Open Access and automatically processable citation data beyond the number alone (32, 33), e.g., to remove self-citations (Figures 11, 12). Data processing and analyses are based on keywords curated in and provided by PubMed; however, these are not double-checked by placebo researchers, or by us. For example, we found some inconsistencies between keywords in the abstracts and MeSH terms that could affect searches and analyses of publications in the field. While both the lags in indexing of new articles, and shortcomings in accuracy of indexing, restrict interpretations concerning the *absolute* number of keywords presented here, our analyses were performed under the assumption that these issues are unsystematic across all fields of research. If true, they would not affect the *relative* numbers between different MeSH and publication types for further analyses. More dedicated analyses would be required for a comprehensive assessment of the issue of misclassification.

Finally, beyond the reasons indicated above, the choice of addiction as a reference field was ultimately arbitrary and there may be more suitable fields, or fields that are of more interest to particular research groups. Further comparisons to evaluate the course of development should (and can easily) be drawn with other research fields. Nevertheless, the choice was meaningful as exemplified by the all but non-existent overlap between the two databases, while simultaneously exhibiting a substantial overlap (36% as per Figure 9B) in terms of the journals in which both fields published. Still, we caution that the methods of obtaining the JIPS and the comparator database were decidedly different, findings therefore have to be viewed with caution.

### Outlook

Prospective developments include the formulation of algorithms for the automated detection of relevant articles, e.g. via machine learning (34, 35). The JIPS database itself is well-suited for this purpose, as it could be contrasted with the corpus of literature (i.e., all PubMed hits for “placebo”, among other sources) from which it is drawn. Another benefit from this endeavor may relate to search engine optimization through recommendation of highly discriminant keywords, as opposed to author- or even expert-indexer-provided keywords.

In summary, the JIPS database is a comprehensive collection of publications in the field of placebo research. Our analyses indicate stable generative capabilities of the field, and an overall performance comparable to the reference field. The methods employed here are easily portable, for example, to identify trends in yet unaddressed subfields. Likewise, the JIPS database itself is available for bibliometric analyses, to address questions to the field or its shortcomings, and to identify blind spots as well as future directions. We invite interested colleagues to use this database for further analyses.

## Data Availability Statement

The raw data supporting the conclusions of this article will be made available by the authors, without undue reservation.

## Author Contributions

PE, KW, and EB did the literature search and assessed data for the JIPS database. PE, BH, and KW provided the study design for this analysis and wrote the first draft. BH and CB analyzed the data and created figures and tables. All authors contributed to the interpretation of results, reviewed and critically revised the manuscript, and approved the final version for submission.

## Funding

This research was supported by the Deutsche Forschungsgemeinschaft (DFG) Collaborative Research Centre/Transregio 289 Treatment Expectation—The impact of expectation on health outcome, Project A02 (Project-ID 422744262–TRR 289).

## Conflict of Interest

The authors declare that the research was conducted in the absence of any commercial or financial relationships that could be construed as a potential conflict of interest.

## Publisher's Note

All claims expressed in this article are solely those of the authors and do not necessarily represent those of their affiliated organizations, or those of the publisher, the editors and the reviewers. Any product that may be evaluated in this article, or claim that may be made by its manufacturer, is not guaranteed or endorsed by the publisher.
