# Supplementary material for: Bibliometric Properties of Placebo Literature From the JIPS Database: A Descriptive Study
Source: Front Psychiatry. 2022 Mar 25;13:853953. doi: 10.3389/fpsyt.2022.853953 (PMC8990029; doi:10.3389/fpsyt.2022.853953)
Supplement: Supplementary file 1 [file Data_Sheet_1.pdf]

Supplementary Information

for

Bibliometric Properties of Placebo Literature from the JIPS Database: A Descriptive Study

Katja Weimer[1*], Cliff Buschhart[2], Ellen K. Broelz[3], Paul Enck[3], Björn Horing[4]

1 Department of Psychosomatic Medicine and Psychotherapy, Ulm University Medical Center, Ulm, Germany

2 University Library, Brandenburg University of Applied Sciences, Brandenburg an der Havel, Germany

3 Department of Psychosomatic Medicine and Psychotherapy, University Hospital Tübingen, Tübingen, Germany

4 Department of Systems Neuroscience, University Medical Center Hamburg-Eppendorf

* corresponding author:

Dr. Katja Weimer

katja.weimer@uni-ulm.de

Supplementary Figure 1. Categorization of publication types in data **(A)**, non-data **(B)**, and uninformative **(C)**.

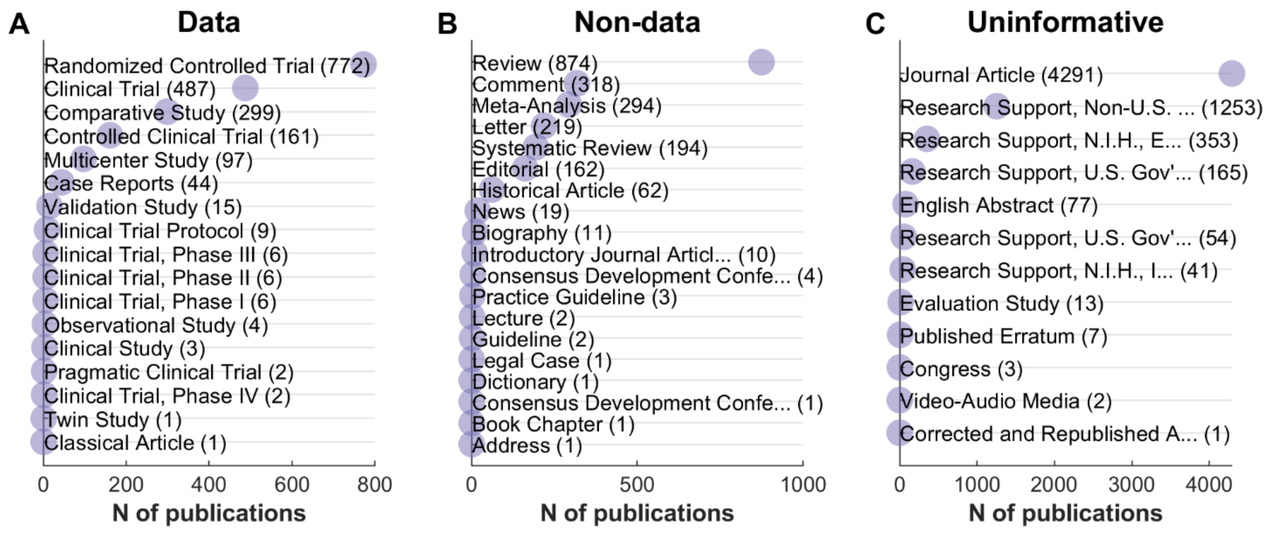

Supplementary Table 1. Non-data publication types considered in Figure 7C and D.

**Panel D category**	**Panel C category**	**Included publication types**
Systematic	Meta-analysis, guideline	Meta-analysis<br>Guideline<br>Practice Guideline<br>Consensus Development Conference<br>Consensus Development Conference, NIH
	Systematic review	Systematic review
Non-systematic	Review	Review
	Other	Address<br>Biography<br>Book Chapter<br>Comment<br>Dictionary<br>Editorial<br>Historical Article<br>Introductory Journal Article<br>Lecture<br>Legal Case<br>Letter<br>News

Supplementary Table 2. Contributions of JIPS or comparator database in journals sorted by 2-year impact factor considered in Figure 9.

**Journal**	**2-year impact factor**	**JIPS contri-butions**	**Addiction contri-butions**
Physiological Reviews	34.32	2	2
Cell	28.45	0	4
Pharmacological Reviews	24.79	2	2
Nature Medicine	23.52	8	2
Nature Genetics	23.1	0	1
Annual Review of Psychology	22.09	2	2

Nature Reviews Genetics	21.22	0	1
New England Journal of Medicine	19.08	24	5
Nature Neuroscience	17.15	3	16
Nature	14.96	8	27
Psychological Bulletin	13.9	6	1
Nature Communications	13.78	3	5
Nature Reviews Gastroenterology and Hepatology	13.14	2	1
Science	13.08	21	27
Gut	12.78	3	0
Annual Review of Medicine	12.74	1	0
Annual Review of Neuroscience	12.47	1	3
Nature Reviews Drug Discovery	12.38	2	0
Journal of Clinical Oncology	12.29	4	2
Trends in Cognitive Sciences	12.01	3	0
Nature Reviews Neuroscience	11.48	6	18
Clinical Psychology Review	11.48	0	23
Diabetes Care	11.38	3	0
Neuron	11.37	7	16
Annals of Oncology	11.13	1	0
Progress in Neurobiology	10.98	4	4
Annual Review of Nutrition	10.69	0	1
Nature Reviews Neurology	10.68	3	0
Nature Reviews Endocrinology	10.39	0	1
Journal of Clinical Investigation	10.27	3	1
Science Translational Medicine	10.13	4	0
Ageing Research Reviews	10.08	0	1
Nature Immunology	10	0	1
Trends in Pharmacological Sciences	9.93	2	3
World Psychiatry	9.57	11	0
Circulation	9.48	3	0
Lancet, The	9.45	43	9
Perspectives on Psychological Science	9.42	2	1
Psychological Methods	9.39	1	0
PLoS Medicine	9.17	5	1
American Economic Review	9.09	0	2
Sleep Medicine Reviews	9.09	1	1
Hepatology	9.04	2	0
Sports Medicine	8.28	3	3
JAMA Psychiatry	8.16	6	10
Diabetologia	8.05	2	0
Science of the Total Environment	7.96	2	0
BMC Medicine	7.86	7	2
Obesity Reviews	7.84	0	5
Annals of Neurology	7.81	3	0
Trends in Neurosciences	7.8	1	11
Trends in Molecular Medicine	7.74	1	4

Psychological Review	7.61	2	0
Drugs	7.6	0	1
JCI insight	7.5	1	0
Brain	7.47	8	3
eLife	7.45	1	0
Journal of the American College of Cardiology	7.44	3	0
Gastroenterology	7.34	11	0
PLoS Biology	7.28	2	0
Nature Reviews Cardiology	7.24	1	0
Drug Discovery Today	7.17	2	0
Molecular Autism	6.87	2	0
Molecular Metabolism	6.86	0	1
Journal of Medicinal Chemistry	6.84	0	2
NeuroImage	6.82	23	1
Journal of Consumer Research	6.69	1	0
Annals of the Rheumatic Diseases	6.55	6	0
Current Neuropharmacology	6.47	1	4
Current Opinion in Neurobiology	6.46	0	8
Journal of Applied Psychology	6.42	3	1
JAMA Neurology	6.37	3	0
European Urology	6.36	2	0
Neurotherapeutics	6.31	2	0
Neuropsychology Review	6.3	0	6
Food Research International	6.3	2	0
Environmental Research	6.28	4	1
Radiology	6.22	1	0
Journal of Behavioral Addictions	6.21	2	312
Current Directions in Psychological Science	6.13	1	0
Journal of Internal Medicine	6.1	2	0
Journal of Gastroenterology	6.1	2	0
Body Image	6.09	0	2
Behavior Research Methods	6.08	0	3
Diabetes	6.07	0	1
Pharmaceutics	6.07	1	0
European Journal of Heart Failure	6.01	1	0
Hypertension	6	6	0
Current Biology	5.99	2	2
Journal of Headache and Pain	5.93	4	2
Journal of Personality and Social Psychology	5.91	5	0
European Respiratory Journal	5.91	5	0
Depression and Anxiety	5.88	1	2
Progress in Cardiovascular Diseases	5.87	1	0
Journal of Abnormal Psychology	5.83	13	4
Clinical Nutrition	5.74	0	2
European Journal of Endocrinology	5.7	1	0
Neuropsychopharmacology	5.66	18	120

Psychological Medicine	5.6	11	5
Computers in Biology and Medicine	5.59	0	1
EBioMedicine	5.57	2	0
Annals of Surgery	5.54	3	0
Journal of Neuroscience	5.54	34	44
International Journal of Molecular Sciences	5.54	0	3
European Heart Journal	5.52	6	0
Neurobiology of Disease	5.44	0	1
Nutrients	5.43	4	51
Food Quality and Preference	5.43	1	0
Child Development	5.38	0	1
Cardiovascular Research	5.35	1	0
Neuroscientist	5.35	0	2
Lancet Public Health, The	5.33	0	3
Frontiers in Pharmacology	5.33	11	0
EMBO Reports	5.32	1	4
Psychological Science	5.32	6	1
Pain	5.32	200	8
Biochemical Pharmacology	5.29	0	2
International Journal of Epidemiology	5.25	1	1
JMIR mHealth and uHealth	5.24	1	2
Tobacco Control	5.23	0	10
Journal of Medical Systems	5.23	0	3
Psychotherapy	5.19	1	0
Acta Pharmacologica Sinica	5.19	0	4
Osteoarthritis and Cartilage	5.17	1	0
Journal of Molecular Biology	5.13	0	1
Journal of Dental Research	5.13	3	0
European Journal of Nutrition	5.06	0	1
Cephalalgia	5.05	23	0
American Journal of Clinical Nutrition	5.02	1	9
Health Psychology Review	5	1	0
